# 539. Impact of a Rapid Diagnostic Assay for the Detection of Bacterial and Viral Agents on Antimicrobial use in Critically Ill Patients with Pneumonia

**DOI:** 10.1093/ofid/ofac492.592

**Published:** 2022-12-15

**Authors:** Lionel K Sielatchom Noubissie, Zachary Ciochetto, Emily Willey, Deanna Olexia, Magdalena Wrzesinski, Blake W Buchan, J Njeri Wainaina, Allison Gibble

**Affiliations:** Yale New Haven Hospital, New Haven, CT; The Medical College of Wisconsin, Milwaukee, Wisconsin; Children's Hospital Wisconsin, Milwaukee, Wisconsin; Froedtert Hospital, Milwaukee, Wisconsin; Froedtert & the Medical College of Wisconsin, Milwaukee, Wisconsin; Medical College of Wisconsin, Milwaukee, Wisconsin; Medical College of Wisconsin, Milwaukee, Wisconsin; Froedtert & the Medical College of Wisconsin, Milwaukee, Wisconsin

## Abstract

**Background:**

Conventional diagnostic methods for pneumonia have a long turnaround time, and a pathogen is isolated in only 33% of cases. As a result, empiric therapy with broad-spectrum antimicrobials often becomes definitive therapy. Rapid molecular diagnostics paired with antimicrobial stewardship intervention have the potential to improve outcomes for patients with lower respiratory tract infections (LRTIs). However, data to support this hypothesis are scarce. This study examined the influence of a rapid multiplex polymerase-chain-reaction (PCR) test on antimicrobial use in critically ill patients with LRTIs.

**Methods:**

This single-center study consisted of pre-implementation (PrIP, December 1, 2018, to February 28, 2019) and post-implementation periods (PoIP, December 1, 2021, to February 28, 2022). Both groups included intensive-care unit (ICU) patients with respiratory cultures from bronchoalveolar lavage (BAL), mini-BAL, or tracheal aspirates. Interventions during the PoIP included concurrent testing of specimens with the BioFire^®^ Pneumonia Panel and clinical decision support tools to streamline treatment recommendations from ICU pharmacists. The primary outcome was the difference in time to optimal therapy (TTOT). Secondary outcomes included time to effective therapy (TTET) and duration of therapy (DOT) for antipseudomonal and anti-MRSA agents.

**Results:**

A total of 163 patients were included (n = 80, PrIP; n = 83, PoIP). The mean age was 57 + 16 years, and medical ICU patients accounted for 60% (n = 97) of cases. The median APACHE II score was the only significant baseline difference between the two arms (22 vs. 18, interquartile range [IQR] 16, 28 vs. 12, 25, p = 0.01). The median TTOT was 38 hours in the PrIP and 21 hours in the PoIP (p < 0.001). TTET was 5.7 hours in the PrIP and 4.9 hours in the PoIP (p = 0.106). The median DOT for antipseudomonal agents decreased by 52% (4.4 days in the PrIP and 2.1 days in the PoIP, p < 0.001), and anti-MRSA agent DOT was 53% shorter in the PoIP (1.9 days vs. 0.9 days, p < 0.001).

**Conclusion:**

Implementation of a rapid multiplex PCR panel for lower respiratory-tract pathogens significantly reduced the time to optimal therapy in critically ill patients with pneumonia. Clinically meaningful reductions in anti-MRSA and anti-pseudomonal agent duration were also noted.

**Disclosures:**

**Blake W. Buchan, PhD**, Accelerate: Advisor/Consultant|Accelerate: Honoraria|BioFire: Honoraria|ChromaCode: Advisor/Consultant|ChromaCode: Honoraria|Pattern: Advisor/Consultant|Pattern: Honoraria.

